# Mitotic regulation by NIMA-related kinases

**DOI:** 10.1186/1747-1028-2-25

**Published:** 2007-08-29

**Authors:** Laura O'Regan, Joelle Blot, Andrew M Fry

**Affiliations:** 1 Department of Biochemistry, University of Leicester, Leicester, UK

## Abstract

The NIMA-related kinases represent a family of serine/threonine kinases implicated in cell cycle control. The founding member of this family, the NIMA kinase of *Aspergillus nidulans*, as well as the fission yeast homologue Fin1, contribute to multiple aspects of mitotic progression including the timing of mitotic entry, chromatin condensation, spindle organization and cytokinesis. Mammals contain a large family of eleven NIMA-related kinases, named Nek1 to Nek11. Of these, there is now substantial evidence that Nek2, Nek6, Nek7 and Nek9 also regulate mitotic events. At least three of these kinases, as well as NIMA and Fin1, have been localized to the microtubule organizing centre of their respective species, namely the centrosome or spindle pole body. Here, they have important functions in microtubule organization and mitotic spindle assembly. Other Nek kinases have been proposed to play microtubule-dependent roles in non-dividing cells, most notably in regulating the axonemal microtubules of cilia and flagella. In this review, we discuss the evidence that NIMA-related kinases make a significant contribution to the orchestration of mitotic progression and thereby protect cells from chromosome instability. Furthermore, we highlight their potential as novel chemotherapeutic targets.

## Background

In 1975, Ron Morris undertook a genetic screen for temperature-sensitive mutants that failed to progress through the cell cycle in the filamentous fungus, *Aspergillus nidulans *[1]. Analysis of the resulting mutants led to some being classified as "bim", as they became blocked in mitosis, while others were called "nim", as they were never in mitosis. The first nim gene to be characterized, nimA, turned out to encode a serine/threonine protein kinase essential for entry into mitosis [2-4]. Mutants arrested in G_2 _when shifted to the restrictive temperature and only entered mitosis upon return to the permissive temperature, while overexpression of wild-type NIMA drove cells into a premature mitosis from any point in the cell cycle [5].

At a similar time, Paul Nurse and Lee Hartwell had undertaken genetic screens for cell division control mutants in fission and budding yeast, respectively, that would ultimately lead to the Nobel Prize in 2001 [6]. Significantly, homologues of NIMA were not identified in these screens and, when they were eventually identified by sequence comparison, the Kin3 kinase in budding yeast and the Fin1 kinase in fission yeast were confirmed as non-essential genes in these organisms [7,8]. At first sight, it therefore appeared that NIMA function might only be required for nuclear division events in the syncitial filamentous type fungi and interest in these kinases remained low-key. However, tantalizing data emerged from the Nurse and Hunter labs in the mid-1990s showing that expression of *Aspergillus *NIMA in fission yeast or vertebrate cells also induced aspects of a premature mitosis, most notably premature chromatin condensation [9,10]. These results were the first evidence that, like other key regulators of the cell cycle, kinases related to NIMA may be important mitotic regulators in higher eukaryotes after all.

**Figure 1:**
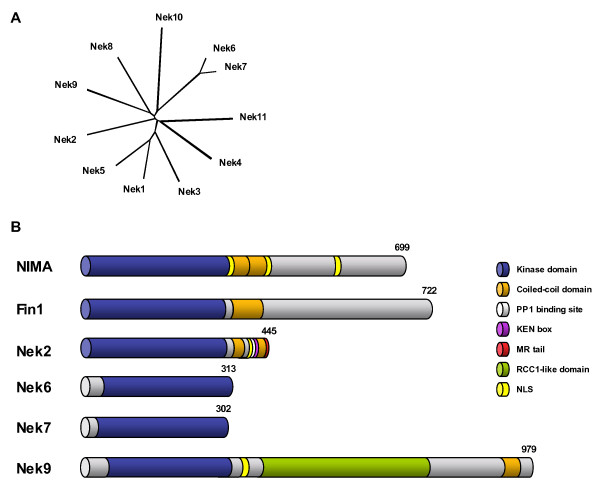
**The NIMA-related kinase family**. **A**. A phylogenetic tree generated by a manually edited multiple sequence alignment of the catalytic domains of the eleven human NIMA-related kinases using the Neighbor Joining method in ClustalX. **B**. A schematic representation of the two fungal (*Aspergillus *NIMA and *S. pombe *Fin1) and four mammalian (Nek2, Nek6, Nek7 and Nek9) NIMA-related kinases implicated in mitotic regulation indicating the relative positions of different domains and motifs. Three splice variants of Nek2 have been described; the longest of these, Nek2A, is shown here. Numbers represent protein length in amino acids.

The first mammalian NIMA-related kinases, Nek1, Nek2 and Nek3, were described by the Pawson and Nigg groups in the early 1990s [11,12]. However, sequencing of the human and mouse genomes unexpectedly revealed the presence of eleven genes that encode a distinct clade of mammalian serine/threonine kinases related to NIMA [13]. Hence, this family, termed Nek1 to Nek11, constitutes approximately 2% of the entire human kinome (Figure 1). These kinases share approximately 40–45% identity with NIMA within their N-terminal catalytic kinase domains, but the C-terminal non-catalytic regions are highly divergent suggesting that each kinase might have a distinct function [14]. Nevertheless, data is now fast emerging that at least four of these kinases, Nek2, Nek6, Nek7 and Nek9, are likely to be important regulators of mitotic progression. In this review, we summarize what is known about the mechanism of action of NIMA and Fin1 in fungal mitoses and then focus on how these four vertebrate kinases might also contribute to cell division.

### NIMA and Fin1 in fungal mitosis

**Figure 2:**
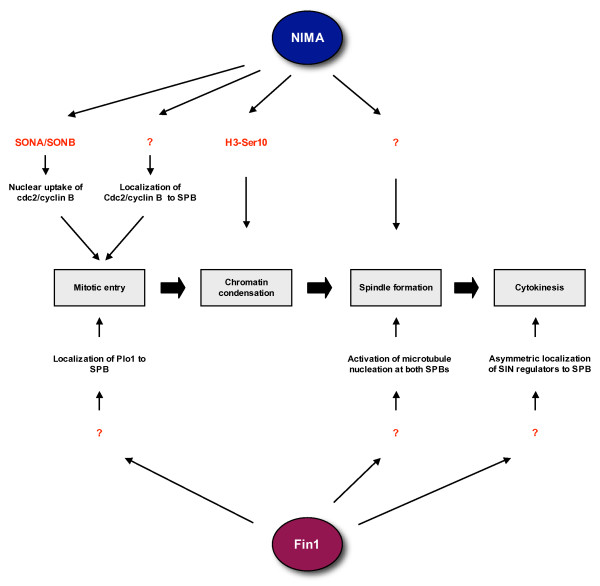
**Regulation of mitotic events by fungal NIMA-related kinases**. The *Aspergillus *NIMA kinase and fission yeast Fin1 kinase regulate multiple events during mitotic progression. Both proteins contribute to the timing of mitotic entry through controlling the localization and/or activation of the Cdc2/cyclin B kinase (Plo1 is an upstream activator of Cdc2/cyclin B). However, the relative importance of NIMA and Fin1 in this event appears to vary as *nimA *mutants block mitotic entry, whereas *fin1 *mutants only delay mitotic entry. Both proteins are also strongly implicated in regulation of mitotic spindle formation. In addition, NIMA promotes chromatin condensation, while Fin1 is involved in the pathway regulating cytokinesis. Unfortunately, the mechanisms by which these kinases operate remain poorly understand primarily because few direct substrates (indicated in red) have been identified.

*Aspergillus *NIMA is essential for mitotic entry and its degradation is necessary for mitotic exit [3,15]. On the other hand, whilst mutations can delay mitotic entry, fission yeast Fin1 is not essential for viability [8,16]. Despite this apparent difference, careful studies on these two fungal kinases have revealed a number of mechanisms by which they might both participate in the control of mitotic entry, chromatin condensation, spindle formation and cytokinesis (Figure 2). NIMA may be essential for mitotic entry as it is required to localize the master mitotic regulator, Cdc2/cyclin B, to the nucleus at this time [17]. Fungi like *Aspergillus *and yeast undertake a closed mitosis in which the nuclear envelope remains intact. Therefore, to initiate chromatin condensation and spindle formation within the nucleus, Cdc2/cyclin B must be translocated to the nucleus. Screening for extragenic suppressors of the *nimA1 *allele led to identification of two components of the nuclear pore complex, SONA, a homologue of yeast Gle2/Rae1, and SONB, a homologue of human Nup98. These genetic interactions suggest that NIMA might directly participate in the nuclear uptake of Cdc2/cyclin B through the nuclear pore [17,18].

NIMA activity is also required to promote localization of Cdc2/cyclin B to the spindle pole body (SPB), the major microtubule organizing centre (MTOC) in fungi and functional equivalent of the higher eukaryotic centrosome [17]. In mammals, Cdk1 (the homologue of Cdc2) is first activated at the centrosome suggesting that concentration of cell cycle regulators at centrosomes/SPBs may be a conserved mechanism for switching on Cdc2/cyclin B activity at the onset of mitosis [19]. In fission yeast, although there is no evidence yet that Fin1 is required to localize Cdc2/cyclin B to either the nucleus or SPB, Cdc2/cyclin B is present on mitotic SPBs [20]. Moreover, Fin1 is required to localize the polo-like kinase, Plo1, to the SPB [16], and Plo1 potentiates the activation of Cdc2/cyclin B via phosphorylation and activation of the intermediary phosphatase, Cdc25 [21]. This would explain why activation of Cdc2/cyclin B and mitotic commitment is delayed in some *fin1 *mutants, even if they eventually enter mitosis, [8,16]. However, in *nimA *mutants arrested in G_2_, Cdc2/cyclin B is fully active and the *Aspergillus *polo-like kinase, PLKA, appears to be correctly localized at the SPB [3,22]. Nevertheless, whilst the molecular pathways may be subtly different, both fungal kinases contribute to the timing of mitotic onset through regulation of Cdc2/cyclin B localization and/or activation. Furthermore, NIMA can be phosphorylated and activated by Cdc2/cyclin B suggesting the existence of a positive feedback loop typical of many pathways regulating cell cycle transitions [23].

Few substrates of NIMA have been identified apart from histone H3 which is phosphorylated by NIMA on Ser-10 [24]. Phosphorylation of H3 Ser-10 is closely correlated with chromatin condensation in many eukaryotes and this could explain why ectopic expression of NIMA drives premature chromatin condensation in yeast, *Xenopus *and human cells [9,10]. However, in most species, phosphorylation of histone H3 on Ser-10, and its close relative CENP-A on the equivalent Ser-7, is primarily executed by the Aurora B kinase [25]. Initial studies suggested that Fin1 overexpression also triggered premature chromatin condensation [8]; however, this was not accompanied by the usual recruitment of condensin and topoisomerase II raising the question of whether this truly reflected a normal mitotic condensation process [26].

Overexpression of NIMA in *Aspergillus *also promotes transient formation of mitotic spindle-like structures [5]. The reason for this remains elusive, although the fact that NIMA and Fin1 localize to mitotic SPBs suggests a potential role in microtubule nucleation, as well as Cdc2/cyclin B activation [17,26,27]. Indeed, an elegant screen for genes required for correct spindle architecture led to the isolation of a temperature-sensitive *fin1 *mutant in which only one of the two SPBs in mitotic cells could nucleate microtubules leading to assembly of monopolar spindles [16]. This caused severe defects in the first mitosis after shift to the restrictive temperature, although cells appeared to adapt to the loss of Fin1 in subsequent divisions. A similar spindle pole phenotype was observed when *Aspergillus nimA *mutants were forced into mitosis by additional mutation of the BIME APC/C subunit [28]. Epistatic interactions with spindle checkpoint mutants further support a requirement for Fin1 in formation of a robust mitotic spindle [16,26]. Yeast two hybrid interactions screens identified *Aspergillus *TINA as a partner of NIMA [29]. TINA specifically localizes to the SPB in mitosis and its deletion leads to excessive nucleation of astral cytoplasmic microtubules during mitosis, albeit without obviously affecting spindle formation. TINA may therefore act to suppress cytoplasmic microtubule nucleation during mitotic progression, although the relative importance of this to nuclear spindle formation and what role NIMA has in controlling TINA function are currently unknown.

In the later stages of mitosis, Fin1 plays a key role in the timing of mitotic exit through modulation of signalling in the septum initiation (SIN) pathway [27]. In a manner analogous to that described above for mitotic entry, many key regulators of the SIN pathway congregate at the SPB, although in this case there is asymmetric concentration to just one, the younger, SPB [30]. In the absence of Fin1 function, SIN regulators are activated on both SPBs enhancing the signal for septation; this implies that Fin1 activity is required to block SIN activation on the older SPB, perhaps to prevent premature septation. Fin1 also associates with the central spindle in late mitosis, although to what purpose is not known [27]. In summary, the exact requirement for NIMA-related kinases in fungal mitoses is likely to vary from organism to organism, but significant overlap is seen in how these enzymes ensure that mitotic events occur with proper timing and fidelity thereby reducing the likelihood of acquiring genetic damage during cell division.

### Nek2: a functional homologue of NIMA?

Of the eleven mammalian Neks, the most closely related by sequence within the catalytic domain to NIMA and Fin1 is Nek2 and, biochemically, NIMA and Nek2 share many common properties [31,32]. These reasons initially led to Nek2 becoming the most closely studied family member in higher eukaryotes [33,34]. Like NIMA and Fin1, Nek2 exhibits a cell cycle-dependent expression and activity profile that strongly suggest a role in mitosis [31]. However, Nek2 does not rescue the phenotypes of *nimA *mutation or Fin1 deletion and so it cannot be considered as the direct homologue [14]. Nevertheless, there are a number of aspects to Nek2 biology that suggest a significant conservation of function.

**Figure 3:**
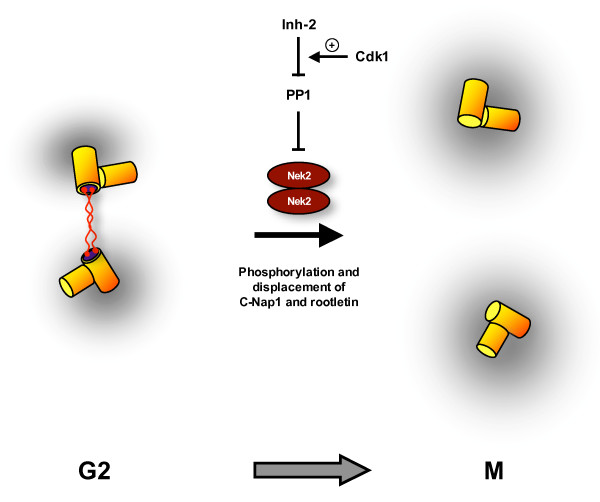
**Nek2 promotes centrosome separation at mitotic onset**. G2 cells contain a duplicated centrosome that consists of two pairs of centrioles (yellow cylinders) surrounded by pericentriolar material (grey cloud). It is proposed that the two centrosomes lie in close proximity as a result of a proteinaceous linker that connects the proximal ends of the parental centrioles. This structure contains at least two proteins, C-Nap1 (blue disc) and rootletin (red fibres). At this time, the Nek2 kinase, which exists as a stable homodimer, is inhibited by the protein phosphatase, PP1. Upon entry into mitosis, PP1 itself is inhibited as a result of binding of the Inhibitor-2 protein (Inh-2), an interaction that may be stimulated by Cdk1. The consequence is that Nek2 kinase is activated leading to phosphorylation and displacement of C-Nap1 and rootletin from the centrosome. The two disconnected pairs of centrioles can then be driven apart to form the two poles of the emerging mitotic spindle.

Nek2 expression is maximal in S and G_2 _of the cell cycle when a major fraction of the protein localizes to the centrosome. Hence, like NIMA and Fin1, Nek2 is a component of the MTOC at the time of mitotic entry. Indeed, Nek2 kinases from *Dictyostelium *and *Drosophila *through to *Xenopus *and humans are localized to centrosomes where they firstly contribute to their structural integrity [35-38]. However, there is little evidence at the present time that Nek2 regulates the timing of mitotic entry by promoting the recruitment of either Plk1 or Cdk1/cyclin B to the centrosome. Instead, it appears to play a more direct role in enabling bipolar spindle formation through initiating the separation of centrosomes at the G_2_/M transition. The first evidence for this came from showing that overexpression of active, but not catalytically-inactive, Nek2 stimulates centrosome separation in interphase cells [35]. Similarly, more recent work showed that RNAi depletion of Nek2 inhibits centrosome separation without significantly affecting mitotic entry [39]. This model was further corroborated by the demonstration that Nek2 interacts with and phosphorylates at least two components of the intercentriolar linkage, C-Nap1 and rootletin [40,41]. This structure acts as a bridge or tether that holds the two centrosomes in close proximity during interphase, but which must be dismantled to allow centrosome separation to occur. Indeed, C-Nap1 and rootletin, which also interact with each other [40,42], are displaced from the centrosome in late G_2 _and thus are absent from mitotic spindle poles. The current working model is that Nek2 phosphorylates C-Nap1 and rootletin triggering dissociation from the centrosome, and possibly each other, leading to loss of centrosome cohesion (Figure 3). This allows centrosomes to be driven apart to the two poles of the emerging mitotic spindle by microtubule-based motor proteins, such as Eg5.

A common feature of the C-terminal regulatory domains of several NIMA-related kinases is the presence of a coiled-coil motif immediately downstream of the catalytic domain. In NIMA, this region is essential for high copy overexpression to cause toxicity suggesting that it is essential to NIMA function [15], while, in Nek2, this coiled-coil acts as a dimerization motif facilitating autophosphorylation and kinase activation [43]. Recent structural studies on Nek2 have identified key sites of autophosphorylation within the catalytic domain [44], and the requirement for autophosphorylation provides a strategy to prevent the premature activation of Nek2, and thus inappropriate separation of centrosomes, in interphase. Protein phosphatase 1 (PP1) binds directly to a KVHF motif in the non-catalytic C-terminal region leading to dephosphorylation and inactivation of Nek2 and, potentially, simultaneous dephosphorylation of associated substrates, such as C-Nap1 and rootletin [45,46]. Conversely, Nek2 can phosphorylate and inhibit PP1. This mutually antagonistic complex acts as an extremely sensitive "bistable switch" that enables a rapid increase in Nek2 autophosphorylation and activation to occur upon inhibition of PP1 by the Inhibitor-2 protein at the onset of mitosis [47].

Ninein-like protein (Nlp) and centrobin are other potential substrates of Nek2 that reside at the centrosome, although, interestingly, Nlp preferentially associates with the older centrosome whereas centrobin asymmetrically associates with the younger centrosome [48,49]. Both are large coiled-coil proteins broadly implicated in the nucleation and/or anchoring of microtubules. Like C-Nap1 and rootletin, endogenous Nlp is displaced from the centrosome upon mitotic onset while recombinant Nlp is displaced by Nek2 overexpression [49]. Centrobin is also displaced from interphase centrosomes upon Nek2 overexpression, but, in untreated cells, centrobin remains present at centrosomes throughout mitosis [50]. These two substrates therefore implicate Nek2 in modification of microtubule organization at the G_2_/M transition. Nlp is also a substrate of the polo-like kinase, Plk1 [51], and, as recruitment of Plk1 to its substrates requires their prior phosphorylation by so-called priming kinases, this raises the question of whether Nek2 is a priming kinase for Plk1 on the substrate Nlp. If it is, then Nek2 could potentially contribute to Plk1 recruitment to the centrosome after all through regulating its interaction with Nlp. This will be an important question to resolve as it could reflect similarity with the function of Fin1 in Plo1 recruitment, which incidentally also requires a coiled-coil protein, Cut12 [16,21].

A part from centrosomal functions, Nek2 may well have other roles in mitotic progression. Reminiscent of the phenotype induced by overexpression of NIMA in higher eukaryotes, murine Nek2 is implicated in chromatin condensation, at least in meiotic spermatocytes. In these cells, there is evidence that Nek2 phosphorylates the chromatin bound protein, HMGA2, downstream of the Erk1/p90^Rsk2 ^pathway [52,53]. Nek2C is a splice variant that exhibits preferential translocation into the nucleus; however, whilst this supports a nuclear function of Nek2, overexpression of Nek2C alone does not induce premature chromatin condensation or phosphorylation of histone H3 in mitotic cells [54]. Nek2 has also been reported to interact with several components of the mitotic checkpoint, most notably Hec1 and Mad1 [55,56]. The significance of these interactions remains unclear as functional studies have not revealed a direct role for Nek2 in mitotic checkpoint signalling. However, suppression or inhibition of Nek2 in early mouse and *Xenopus *embryos led to abnormal spindle structures and abortive cleavages that could reflect both centrosomal and mitotic checkpoint defects [37,57].

Finally, it is conceivable that, as for Fin1, Nek2 could have a role in late mitosis/cytokinesis. Nek2A, the most abundant splice variant, is degraded in an APC/C-dependent manner after mitotic entry in adult cells [58,59]. However, in early mouse embryos, Nek2A is not degraded and localizes to the midbody in late mitosis [57]. Likewise, Nek2B, a splice variant that lacks the C-terminal destruction signals, is present in late mitosis in adult cells and specific depletion of Nek2B has been reported to delay mitotic exit [39]. Nek2 is also detected at the midbody in late mitosis in *Drosophila *where overexpression led to mislocalization of actin and anillin, and ectopic sites of cleavage furrow formation [38]. However, substrates of Nek2 remain to be identified that would provide a mechanistic explanation for a role in late mitosis.

### Nek9 and mitotic spindle formation

The second mammalian Nek to be directly implicated in mitotic progression was Nek9, also called Nercc1 [60] and, in one earlier report, Nek8 [61]. Nek9 is one of the longer Neks with a relatively large C-terminal non-catalytic domain that contains an RCC1 homology region and, like Nek2, a coiled-coil dimerization motif [14]. Evidence for a role in mitosis has come from observing its cell cycle-dependent activity and localization, functional experiments and identification of interacting partners.

The expression of Nek9 protein remains constant through the cell cycle [60,61]. However, its kinase activity, like that of NIMA, specifically increases in mitosis [60]. This activation depends upon the mitotic-specific phosphorylation of T210 in the activation loop. Whether this is an autophosphorylation event is unclear, but mutants lacking the coiled-coil dimerization motif have significantly reduced activity. Interestingly, deletion of the RCC1 domain leads to a hyperactive kinase indicating that this region may be required for autoinhibition of Nek9 [60]. The protein sequences of both *Aspergillus *NIMA and vertebrate Nek9 contain a number of potential Cdk1 phosphorylation sites; however, it remains to be proven whether either enzyme is a substrate of Cdk1/cyclin B in vivo. In terms of localization, both endogenous and recombinant full-length Nek9 are diffusely distributed within the cytoplasm in interphase and mitosis [60-62]. However, a number of Nek9 mutants localize to the nucleus in certain cell types suggesting that a nuclear localization sequence, immediately downstream of the catalytic domain, is functional but tightly regulated [60,62]. More pertinently, generation of a phosphospecific antibody against the phospho-T210 site revealed concentration of activated Nek9 on spindle poles during mitosis [63]. Similarly, *Xenopus *Nek9 was detected on the poles of spindles assembled in vitro in frog egg extracts [63]. Hence, although the bulk of the protein may be soluble in the cytoplasm, a fraction of the protein may be tightly associated with the centrosome where it can be specifically activated, possibly by Cdk1/cyclin B, at the onset of mitosis [19].

Functional experiments have also implicated Nek9 in mitotic progression and, specifically, in spindle organization. First of all, expression of wild-type and kinase-inactive Nek9 proteins in human cells led to a reduced mitotic index and an accompanying increase in apoptosis, while expression of constructs that localize to the nucleus led to micronucleated and multinucleated cells, phenotypes that are often associated with defective mitoses [60]. Secondly, injection of anti-Nek9 antibodies into human cells appeared to block mitotic entry. However, more recent RNA interference experiments in which almost complete depletion of Nek9 in human cells was achieved did not lead to any major alteration in cell cycle progression [62]. In agreement with this, immunodepletion of Nek9 from *Xenopus *egg extracts did not prevent cell cycle progression *in vitro *[63]. Nevertheless, while Nek9 may not be essential for mitotic entry, there is clear evidence that it is necessary for the accurate segregation of chromosomes. Cells injected with anti-Nek9 antibodies after mitotic entry accumulated defects in mitotic spindle formation and chromosome segregation. Furthermore, depletion experiments in *Xenopus *egg extracts revealed problems with both bipolar spindle formation from sperm chromatin and microtubule aster formation induced by addition of a constitutively active Ran GTPase mutant (Q69L) [63]. Together, these data imply that Nek9 may contribute to both centrosomal (sperm chromatin) and acentrosomal (Ran-Q69L) pathways of spindle formation, although the precise mechanisms of action remain obscure.

The presence of the RCC1-like domain and the effect on Ran-mediated aster formation in vitro raised the strong possibility that Nek9 might regulate spindle formation via the Ran GTPase. RCC1 is a guanine nucleotide exchange factor (GEF) that stimulates Ran-GTP formation. In mitosis, RCC1 is concentrated on condensed chromatin creating a gradient of Ran-GTP that increases towards the DNA. Ran-GTP binds to importin-α triggering release of spindle promoting factors, such as TPX2, in the vicinity of the chromatin [64]. Binding experiments indicated that Ran does indeed bind to Nek9 via the RCC1 domain as well as, surprisingly, its catalytic domain [60]. However, whereas RCC1 is an active GEF, the RCC1-like domain of Nek9 lacks some of the critical amino acids required for such activity suggesting that Nek9 may not function as a GEF, although this has yet to be directly tested [61]. Equally, the interaction between Ran GTPase and Nek9 does not seem to regulate Nek9 activity. Hence, whilst the presence of this domain, the interaction with Ran and the regulation of Ran-induced microtubule aster formation are intriguing, it remains to be determined how Nek9 and Ran cooperate in spindle organization.

Other microtubule and centrosome related proteins have been identified as partners for Nek9 that shed further light on its putative spindle function; these include Bicaudal-D and γ-tubulin. The Bicaudal-D2 isoform was identified as a partner and substrate of Nek9 following copurification of the two proteins from rabbit lung [61]. Bicaudal-D proteins are involved in dynein-mediated microtubule-dependent transport and have recently been implicated in microtubule anchoring at the centrosome [65]. Meanwhile, immunoprecipitates of Nek9 from *Xenopus *egg extracts contained γ-tubulin and other members of the γ-tubulin ring complex [63]. This key component of the centrosome is absolutely essential for microtubule nucleation. Hence, these interactions, together with the localization data, suggest that Nek9, like Nek2, may directly regulate centrosomal microtubule anchoring and/or nucleation during spindle assembly.

Consistent with the localization of certain Nek9 mutants to the nucleus, Nek9 has been reported to interact with the 600 kDa chromatin-associated complex, FACT, that facilitates polymerase progression during DNA transcription and replication [66], and with the adenoviral nuclear oncoprotein, E1A [62]. The interaction with FACT and the detection of a minor G_1_/S delay upon Nek9 depletion was used to imply a potential role in G_1_/S progression [66]. However, this remains to be substantiated as other RNAi studies showed no defect in cell cycle progression [62]. The association with E1A appears to stimulate removal of Nek9 from the nucleus and, as most viral oncoproteins promote cell cycle progression, this would argue that nuclear Nek9 may somehow act as a negative regulator of the cell cycle. Clearly, the relevance and purpose of these interactions requires further study.

**Figure 4:**
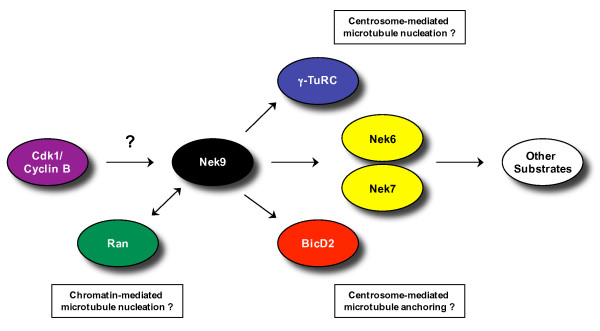
**Mitotic interactions of the Nek9 kinase**. This figure illustrates what is currently known about the potential upstream regulation and downstream targets of the Nek9 kinase during mitosis. It also suggests possible mechanisms by which Nek9 might regulate spindle organization, although these currently remain only hypotheses. Cdk1 can phosphorylate Nek9 in vitro and both proteins localize to the centrosome; hence, it is possible that Nek9 is activated upon mitotic entry by Cdk1/cyclin B. Once active, it is attractive to speculate that Nek9 may regulate chromatin-mediated microtubule nucleation as Nek9 can interact with Ran through its RCC1-like and catalytic domains. However, it is yet to be shown whether Nek9 influences Ran activity or vice versa. Equally, Nek9 may regulate centrosome-mediated microtubule nucleation and/or anchoring via its interactions with the γ-tubulin ring complex (γ-TuRC) and BicD2, respectively. Finally, Nek9 is proposed to activate two other NIMA-related kinases, Nek6 and Nek7, and, although substrates of Nek6 and Nek7 remain to be identified, it is likely that these also play important roles in mitotic spindle organisation and chromosome segregation.

Finally, Nek6, and by analogy the closely-related Nek7, have also been established as partners and substrates of Nek9, binding to the region of Nek9 between the RCC1-like domain and the coiled-coil motif [60]. Although the interaction between Nek6 and Nek9 is stable, assays with various mutants seem to indicate that Nek6 needs to be active for this interaction to take place, whereas Nek9 does not [67]. Furthermore, the C-terminal region of Nek9 can act as a negative regulator of Nek6 and Nek7 activity. It is therefore proposed that Nek9 acts upstream of Nek6 and Nek7 phosphorylating and activating these kinases specifically in mitosis (Figure 4). This is discussed in more detail in the following section.

### Nek6 and Nek7: targets of the Nek9 kinase

Nek6 and Nek7 represent the final two NIMA-related kinases implicated in mitotic progression to date. They are the smallest and, structurally, the simplest NIMA-related kinases, consisting solely of a catalytic domain without the C-terminal regulatory domain common among other Neks [14]. Nek6 and Nek7 share 87% amino acid identity within the kinase domain and only significantly differ from one another within a short N-terminal extension. These protein kinases, along with the related F196H6.1 from *C. elegans*, have therefore been suggested to represent a highly conserved subfamily of the NIMA-related kinases [68]. Nek6 and Nek7 were initially identified in a classic biochemical screen for kinases capable of phosphorylating the hydrophobic regulatory site of the p70 ribosomal S6 kinase (S6K) [69]. This suggested a role in growth factor signalling that was at variance with the role of NIMA in mitotic control. Subsequently, though, it was shown that despite its ability to phosphorylate and activate S6K *in vitro*, S6K was unlikely to be a physiological substrate of Nek6 [70], and more recent evidence supports a NIMA-like mitotic role for both Nek6 and Nek7.

Endogenous Nek6 is activated during mitosis, concomitant with an increase in Nek6 protein level [67]. Nek7 protein, on the other hand, appears to be relatively constant throughout the cell cycle [71]. Importantly, Nek7 has been reported to localise to the centrosome in both interphase and mitotic U2OS and HeLa cells [71,72]. Thus, in common with other NIMA-related kinases implicated in mitotic regulation, Nek7 is localised to the MTOC at the time of mitotic entry. However, whereas the presence of Nek2 at the centrosome promotes spindle pole separation at the G_2_/M transition, Nek7, like Nek9, may be more directly involved in nucleation and organisation of spindle microtubules through recruitment of γ-tubulin and its partners. siRNA knockdown of Nek7 results in decreased levels of centrosomal γ-tubulin and a diminished microtubule nucleating activity [71], although to date there is no evidence to support an interaction between Nek6 and/or Nek7 with γ-tubulin or its partners. Indeed, Nek6 has not been shown to localise to the centrosome either at interphase or mitosis; rather, it has a diffuse, mainly cytoplasmic, localisation with no strong association to any mitotic structures. Nevertheless, overexpression of catalytically-inactive Nek6 and Nek7 does produce similar phenotypes. Typically, cells exhibit increased mitotic indices, spindle defects, nuclear abnormalities and apoptosis [72,73]. These phenotypes are also seen upon RNAi knockdown of either Nek6 or Nek7 in HeLa cells [71-73]. Cells frequently arrest at mitosis with normal chromatin condensation and alignment but are unable to complete chromosome segregation. Therefore, Nek6 and Nek7 activity appears to be required for proper anaphase progression with cells either arresting at the spindle checkpoint and undergoing apoptosis, or completing mitosis but with the acquisition of nuclear abnormalities in the process. As HeLa cells treated with Nek6 or Nek7 siRNAs or overexpressing catalytically-inactive Nek6 or Nek7 show significant numbers of abnormal mitotic spindles, and given the centrosomal localisation of Nek7 and reduced microtubule nucleating activity of Nek7 depleted cells, it seems likely that these kinases exert their effects through organisation of the mitotic spindle.

As previously mentioned, Nek6 and Nek7 copurify with Nek9 as a result of a specific interaction with a region of the Nek9 protein close to, but distinct from, its coiled-coil motif. Nek9 can then stimulate Nek6 and Nek7 activity through phosphorylation of the Ser^206 ^and Ser^195 ^residues within the activation loop of Nek6 and Nek7, respectively [67]. This has led to the working model that Nek6, Nek7 and Nek9 act together in a novel mitotic cascade in which Nek9, maintained in an inactive state during interphase, is activated at mitosis resulting in the activation of its downstream substrates, Nek6 and Nek7. In turn, these two kinases coordinate the formation and/or maintenance of the mitotic spindle. This model is supported by the similarity in defects that arise from interference of either Nek9 or Nek6/Nek7. However, Nek7 is found not only found at the centrosome during mitosis, but also the spindle midzone and midbody of late mitotic cells [71]. Simultaneous knockdown of both Nek7 and MAD2, in order to circumvent the spindle checkpoint and therefore reduce the metaphase arrest phenotype, results in a failure of cells to undergo cytokinesis. Hence, Nek7 may, in common with Fin1 and perhaps also Nek2, have an additional role in late mitosis/cytokinesis.

Many questions clearly remain to be resolved concerning this putative mitotic Nek cascade. For example, does Nek9 have additional substrates besides Nek6 and Nek7 or are all of its functions executed through these two kinases. Likewise, no substrates of Nek6 and Nek7 have been identified to date, although the most obvious candidates would be spindle or centrosome components. Another question is whether Nek6 and Nek7 have entirely redundant roles or whether their distinct N-termini allow differences in their regulation or function. Whilst interfering with endogenous Nek6 and Nek7 by overexpression of catalytically-inactive mutants or RNAi knockdown results in similar phenotypes, there are reported differences in their cell cycle-dependent activity and localisation. Furthermore, whilst the two kinases exhibit largely complementary patterns of tissue expression, they apparently respond differently under conditions of serum deprivation: Nek7 is rapidly activated by serum deprivation, whereas Nek6 is inhibited [74].

Nek6 has also been shown to interact with the peptidyl-prolyl isomerase, Pin1, in the hepatic carcinoma cell line, Hep3B [75]. Pin1 is not only essential for cell cycle progression in yeast and mammalian cells, but its depletion produces a similar mitotic arrest/apoptosis phenotype as seen upon depletion of Nek6 and Nek7. Pin1 overexpression is prevalent in human cancers, including liver cancer, and upregulation of Nek6 mRNA was found to correlate with Pin1 upregulation in 70% of hepatic cell carcinomas [75]. This then provides the first evidence tentatively linking Nek6 to carcinogenesis and, together with the fact that overexpression of catalytically-inactive Nek6 reduces the growth rate of MDA-MB-231 human breast cancer cells [73], highlights Nek6 as a potential chemotherapeutic target.

### Conserved roles for Neks in microtubule organization

So far, then, of the eleven human Nek kinases, only Nek2, Nek6, Nek7 and Nek9 have been shown to function in mitotic regulation. However, a more general theme that is emerging for the function of many, and perhaps all, Neks is the organization of microtubules. It is clear from the discussion above that certain NIMA-related kinases from different organisms regulate microtubules during mitosis. What was not expected from early studies in the non-ciliated unicellular fungi, *Aspergillus *and fission yeast, was that some Neks may also have important functions in microtubule organization within cilia [76]. It is now apparent that the biology of centrosomes, cilia and cell cycle control are closely linked: in dividing cells, centrosomes, which are composed of two barrel-shaped centrioles surrounded by pericentriolar material, organize the cytoplasmic microtubule network and the mitotic spindle; however, when cells stop dividing, the centrioles migrate to the cell surface where they nucleate the microtubules of the ciliary, or flagellar, axoneme [77]. In the latter context, centrioles are referred to as basal bodies.

The first suggestion of a Nek function in cilia emerged from studies performed in ciliated unicellular eukaryotes, namely *Chlamydomonas *and *Tetrahymena*. *Chlamydomonas *is a biflagellate and encodes two Neks: Fa2p, which is required for microtubule severing and deflagellation as cells progress towards mitosis, and Cnk2p, which regulates both flagellar length and cell size [78,79]. *Tetrahymena *has hundreds of cilia, which fall into distinct classes depending on their localization and length. Amazingly, this organism has 39 nek genes and all of the encoded proteins studied so far localize to cilia. Here, they may determine individual cilia length, as overexpression of wild-type kinases reduces cilia length while overexpression of kinase-inactive mutants increases cilia length [80].

Of the mammalian Neks, both Nek1 and Nek8 were unexpectedly implicated in cilia function when it was found that mutations in laboratory mouse models of polycystic kidney disease (PKD) mapped to the Nek1 and Nek8 genes [81,82]. PKD is a classic indication of an underlying ciliopathy and so this discovery raised the prospect that mammalian Neks may also have a role in microtubule organization in cilia. Indeed, antisense Nek8 oligonucleotides induce pronephric cysts in Zebrafish with depletion of Nek8 leading not to loss of cilia formation but to longer cilia [81,83]. This is consistent with the role of *Tetrahymena *Neks in regulating cilia length. Nek8 is localized to the proximal region of the primary cilia, implying that Nek8 could contribute to microtubule turnover at this end of the ciliary axoneme [84].

Nek8, though, is most closely related to Nek9, which clearly functions in mitosis, while overexpression of Nek1 has been reported to induce premature chromatin condensation in a manner not dissimilar to overexpression of NIMA [85]. Furthermore, endogenous Nek1 localizes to centrosomes in interphase and mitosis, and proteins involved in the G_2_/M DNA damage checkpoint, as well as microtubule-dependent transport and PKD, were identified as two hybrid partners of Nek1 [84,86]. Thus, there is evidence in support of both cell cycle and ciliary functions for these two Nek kinases. Pertinent to this review, cilia are important signalling organelles that can influence the state of proliferation of a cell [87]. Indeed, aberrant signalling from defective cilia is likely to contribute to the overproliferation of kidney cells that lead to renal cyst formation. Hence, an exciting possibility is that Nek kinases act in signalling pathways that determine cell fate with respect to differentiation and mitotic proliferation.

### Future Perspectives

Protein kinases involved in cell cycle progression or growth related signalling pathways make attractive targets for the development of novel anti-cancer agents. Small molecule inhibitors have already been identified in high throughput screens against the Cdk1, Aurora A and B, and Plk1 mitotic kinases and these are under further development for potential clinical use [88]. As it now becomes apparent that members of the human NIMA-related kinase family also play important roles in mitotic progression, these will serve as a further rich source of potential new targets [89]. Like Nek6, Nek2 is also upregulated in various cancer cell lines, as well as primary breast tumours [90-92], and the hope is that interfering with mitotic Neks may arrest cell growth or promote apoptosis in a tumour-specific manner. It will also be important to determine whether specific inhibitors can be developed against the different Nek kinases and which of these kinases makes the most effective target. Clearly, the exploitation of these kinases as novel drug targets requires a better understanding of their basic biology than we have at the present time. For example, it will be important to determine whether Nek9, Nek6 or Nek7 regulates microtubule nucleation at spindle poles through association or phosphorylation of γ-tubulin-associated proteins, or whether they regulate stability of spindle microtubules through a Ran-mediated mechanism. In particular, further substrate identification is key to unravelling the mitotic functions of Nek6, Nek7 and Nek9. Nevertheless, with their therapeutic potential in mind, it would appear that NIMA-related kinases are at long last stepping into the limelight.

## Competing interests

The author(s) declare that they have no competing interests.

## Authors' contributions

All authors contributed to the conception and writing of this article and have read and approved the final manuscript.
